# Quantitative Genetics of Genomic Imprinting: A Comparison of Simple Variance Derivations, the Effects of Inbreeding, and Response to Selection

**DOI:** 10.1534/g3.111.000042

**Published:** 2011-07-01

**Authors:** Anna W. Santure, Hamish G. Spencer

**Affiliations:** * Allan Wilson Centre for Molecular Ecology and Evolution, University of Otago, Dunedin, New Zealand; ‡ National Research Centre for Growth and Development, University of Otago, Dunedin, New Zealand; † Department of Zoology, University of Otago, Dunedin, New Zealand

**Keywords:** breeder’s equation, correlations between relatives, breeding value, additive effect, parental effect

## Abstract

The level of expression of an imprinted gene is dependent on the sex of the parent from which it was inherited. As a result, reciprocal heterozygotes in a population may have different mean phenotypes for quantitative traits. Using standard quantitative genetic methods for deriving breeding values, population variances, and covariances between relatives, we demonstrate that although these approaches are equivalent under Mendelian expression, this equivalence is lost when genomic imprinting is acting. Imprinting introduces both parent-of-origin-dependent and generation-dependent effects that result in differences in the way additive and dominance effects are defined for the various approaches. Further, imprinting creates a covariance between additive and dominance terms absent under Mendelian expression, but the expression for this covariance cannot be derived using a number of the standard approaches for defining additive and dominance terms. Inbreeding also generates such a covariance, and we demonstrate that a modified method for partitioning variances can easily accommodate both inbreeding and imprinting. As with inbreeding, the concept of breeding values has no useful meaning for an imprinted trait. Finally, we derive the expression for the response to selection under imprinting, and conclude that the response to selection for an imprinted trait cannot be predicted from the breeder’s equation, even when there is no dominance.

A gene is imprinted when its level of expression is dependent on the sex of the parent from which it was inherited. For example, insulin-like growth factor 2 (*Igf*2) is expressed only from the paternal allele in most fetal tissues of eutherian and marsupial mammals, while the maternally inherited allele is inactivated (DeChiara *et al.* 1991; O’Neill *et al.* 2000). More generally, imprinting results in nonequivalence of reciprocal heterozygotes, where inheriting an *A*_1_ allele from one’s mother and an *A*_2_ allele from one’s father gives a different phenotype, on average, than the reverse inheritance pattern. Complex processes of epigenetic regulation are necessary for the repression of one allele while the other is expressed. These processes include allele-specific modifications such as differential DNA methylation, chromatin structure and histone packing, and differences in replication timing of the maternally and paternally inherited genomes (Rand and Cedar 2003).

Approximately 234 imprinted genes have been identified in mammals, including 68 in humans, and many of these genes are thought to be involved in traits such as growth and development (Morison *et al.* 2005). A publication predicting imprinted genes based on sequence characteristics suggests that imprinted loci in the human genome number as high as 156 (Luedi *et al.* 2007). Recent years have seen an increasing number of statistical methods developed that aim to identify imprinting in quantitative traits. Using QTL mapping, for example, imprinting has been suggested for quantitative traits as diverse as carcass composition, growth, coat color and reproductive traits (de Koning *et al.* 2001; de Koning *et al.* 2000; Hager *et al.* 2009; Hirooka *et al.* 2001; Knott *et al.* 1998; Lee *et al.* 2003; Milan *et al.* 2002; Quintanilla *et al.* 2002; Rattink *et al.* 2000), while general mixed models have demonstrated the involvement of imprinting in traits such as milk yield, litter size, and growth (de Vries *et al.* 1994; Engellandt and Tier 2002; Essl and Voith 2002; Kaiser *et al.* 1998; Schaeffer *et al.* 1989; Stella *et al.* 2003; Tier and Solkner 1993).

The inclusion of imprinting effects in these genetic methods highlights the significance of imprinting to a range of economically important livestock production traits and to human health and disease, as well as the importance of understanding the effect imprinting may have on traditional approaches to modeling quantitative genetic traits. Quantitative traits may be influenced by many genes, the environment, and any number of interactions between them, and models for these traits are correspondingly complex. Nevertheless, we here employ a one-locus, two-allele quantitative genetic model to demonstrate the differences in a number of standard approaches for theoretically defining breeding values, genotypic variance and covariances between relatives. In doing so, we show that genomic imprinting may have a significant effect on the assumptions made in these most minimal models, and is therefore likely to also influence more complex models involving many alleles and multiple genetic loci. We also compare the effects of imprinting and inbreeding on quantitative genetic parameters and predict the response to selection for an imprinted trait.

In Table 1, we list and define the important symbols used in this paper, following the convention of Nagylaki and Lou (2007). The reference is either to the equation closest to the definition of each symbol [thus (7), (7)+, and (7)- would mean equation (7), the text below (7), and the text above (7), respectively], or to or the relevant approach or table. The table is ordered alphabetically and split into Roman letters, Greek letters, equation simplifications, and subscripts and superscripts.

**Table 1:** Important symbols used in this paper

Symbol	Reference	Definition
Roman letters		
* a*	(1)−	Additive term
* A_i_*	(1)−	Allele *i*
* E*	(19)+	Expectation
* f*	(5)−	Inbreeding coefficient
* G_ij_*	(1)−	Genotypic value of genotype *A_i_A_j_*
* h*^2^	(33)+	Narrow sense heritability
* k*	(9)−	Dominance term
* k_i_*	(1)−	Imprinting term
* p_i_*	(1)−	Frequency of allele *i*
* S*	(19)+	Selection differential
* t*	(18)+	Selection coefficient
* w_ij_*	(18)+	Relative fitness of genotype *A_i_A_j_*
Greek letters		
Δμ	(19)−	Response to selection
δ	(20)−	Difference between the mean genotypic values of offspring before and after selection
ε_*i*_	Approach 3a	Average additive effect of allele *A_i_*
$\varepsilon_{i•}$	Approach 2a	Average additive effect of inheriting an *A_i_* allele from the mother
$\varepsilon_{•j}$	Approach 2a	Average effect of inheriting an *A_j_* allele from the father
φ_*ij*_	(18)−	Absolute fitness of genotype *A_i_A_j_*
$\overset{-}{\varphi }$	(18)+	Mean fitness
λ_*ij*_	Approach 2a	Dominance effect of genotype *A_i_A_j_*
μ	(1)−	Population mean
$\sigma_{G}^{2}$	(2)−	Total genetic variance
$\sigma_{A}^{2}$	Table 3	Additive variance
$\sigma_{D}^{2}$	Table 3	Dominance variance
$\sigma_{AD}$	Table 3	Covariance between additive and dominance effects
Equation simplifications		
α	(10)	$a(1+k(p_{1}-p_{2}))$
α_*f*_	(3)	$a(1+k_{1}p_{1}-k_{2}p_{2})$
α_*m*_	(4)	$a(1+k_{2}p_{1}-k_{1}p_{2})$
γ	(30)	$\frac{1}{2}(\sigma_{Af(1)}^{2}+\sigma_{ADf(1)}+\sigma_{Am(1)}^{2}+\sigma_{ADm(1)})$
ψ	(29)	$\sqrt{\sigma_{D(1)}^{2}+\sigma_{ADf(1)}+\sigma_{ADm(1)}}$
Subscripts and superscripts		
* f*	(12)+	Female (maternal)
* I*	(5)−	Inbreeding model
* ij*	(1)−	Genotype *A_i_A_j_*
* m*	(12)+	Male (paternal)
*	(25)−	Next generation before selection

## The Model

**Figure 1.**
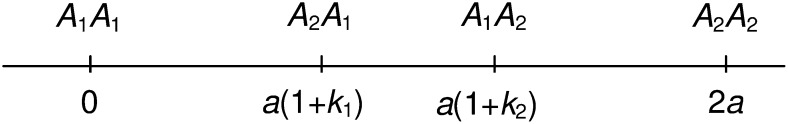
Genotypic values (*G_ij_*) for genotypes *A_i_A_j_* under genomic imprinting.

We here present an overview of a number of approaches for deriving quantitative genetic models for imprinting at one locus. Such models are the basis for many quantitative genetic approaches for dissecting genetic and environmental effects in quantitative traits. Following the approach of Spencer (2002), consider an autosomal diallelic locus subject to imprinting, with alleles *A*_1_ and *A*_2_ at frequency *p*_1_ and *p*_2_ (= 1 − *p*_1_) respectively in the population. Note that the population under consideration is static, without selection, migration or mutation operating. Assume that on some suitable scale, the genotypic value (*G_ij_* for genotype *A_i_A_j_*) of *A*_1_*A*_1_ homozygotes is 0 and *A*_2_*A*_2_ homozygotes is 2*a*. Assuming no maternal effects, writing the maternally inherited allele first, *A*_2_*A*_1_ heterozygotes have genotypic value *a*(1+*k*_1_) and *A*_1_*A*_2_ heterozygotes have value *a*(1+*k*_2_), following the notation of Santure and Spencer (2006) (Figure 1).

In general, imprinting is thought of as complete inactivation of one allele dependent on parental origin, corresponding to *k*_1_ = −1 and *k*_2_ = 1 (complete silencing of the maternal allele), or *k*_1_ = 1 and *k*_2_ = −1 (complete silencing of the paternal allele). More recently, however, imprinting has been treated as a quantitative trait, which implies that maternal or paternal alleles may only be partially inactivated (see, *e.g.*, Naumova and Croteau 2004; Sandovici *et al.* 2005; Sandovici *et al.* 2003), and *k*_1_ and *k*_2_ may take any values in the range [−1,1]. Note that if *k*_1_ = *k*_2_ = 0 then the trait is purely additive, and both reciprocal heterozygotes have a genotypic value midway between the homozygotes. If *k*_1_ and *k*_2_ are equal but of opposite sign (for example, *k*_1_ = −1 and *k*_2_ = 1, giving complete maternal inactivation) then the locus is subject only to imprinting. However, in the most general case, where *k*_1_ and *k*_2_ take any values in the range [−1,1], we might consider that both imprinting and dominance are acting on the locus, as the mean genotypic value of heterozygotes is not the mean of the homozygotes.

With the help of Figure 1, the mean genotypic value over the population is

$$\begin{array}{c} \mu =p_{1}^{2}(0)+p_{2}p_{1}(a(1+k_{1}))+p_{1}p_{2}(a(1+k_{2}))+p_{2}^{2}(2a)\\ =ap_{2}(2+p_{1}(k_{1}+k_{2})). \end{array}$$ (1)

and the total genetic variance is

$$\sigma_{G}^{2}=p_{1}p_{2}(\alpha_{f}^{2}+\alpha_{m}^{2}+a^{2}p_{1}p_{2}{\left(k_{1}+k_{2}\right)}^{2})$$ (2)

where

$$\alpha_{f}=a(1+k_{1}p_{1}-k_{2}p_{2})$$ (3)

and

$$\alpha_{m}=a(1+k_{2}p_{1}-k_{1}p_{2}).$$ (4)

(Spencer 2002). We follow a number of approaches in calculating breeding values, components of variance and covariances between relatives. Doing so illustrates that various assumptions made in these approaches are not valid in the presence of imprinting.

## Approaches

Five approaches are outlined in the Appendix, and the results of their partitioning breeding values, the corresponding calculation of variances and covariances, and the derivation of covariances between relatives are shown in Tables 2–4. In the absence of imprinting, all of these approaches give identical breeding values, variance components, and covariances between relatives. The expressions for these terms in the absence of imprinting are obtained by setting *k*_1_ = *k*_2_ = *k*. Importantly, it can be seen that it is only by modifying the least squares regression approach (Approach 2b) can the sex-specific additive and dominance values derived by Spencer (2002) be recovered (Santure and Spencer 2006). The other three approaches (Approaches 2a, 3a, and 3b) fail to incorporate sex effects, and give incorrect results when partitioning the variance components and calculating covariances between relatives.

**Table 2:** Summary of breeding values for all approaches

	Genotype			
	$A_{1}A_{1}$	$A_{2}A_{1}$	$A_{1}A_{2}$	$A_{2}A_{2}$
Approach 1 and 2b				
Female	$-2p_{2}\alpha_{f}$	$\alpha_{f}(p_{1}-p_{2})$	$\alpha_{f}(p_{1}-p_{2})$	$2p_{1}\alpha_{f}$
Male	$-2p_{2}\alpha_{m}$	$\alpha_{m}(p_{1}-p_{2})$	$\alpha_{m}(p_{1}-p_{2})$	$2p_{1}\alpha_{m}$
Mean	$-p_{2}(\alpha_{f}+\alpha_{m})$	$\frac{1}{2}(p_{1}-p_{2})(\alpha_{f}+\alpha_{m})$	$\frac{1}{2}(p_{1}-p_{2})(\alpha_{f}+\alpha_{m})$	$p_{1}(\alpha_{f}+\alpha_{m})$
		$\text{mean}=\frac{1}{2}(p_{1}-p_{2})(\alpha_{f}+\alpha_{m})$		
Approach 2a	$-p_{2}(\alpha_{f}+\alpha_{m})$	$p_{1}\alpha_{f}-p_{2}\alpha_{m}$	$-p_{2}\alpha_{f}+p_{1}\alpha_{m}$	$p_{1}(\alpha_{f}+\alpha_{m})$
		$\text{mean}=\frac{1}{2}(p_{1}-p_{2})(\alpha_{f}+\alpha_{m})$		
Approach 3a	$-p_{2}(\alpha_{f}+\alpha_{m})$	$\frac{1}{2}(p_{1}-p_{2})(\alpha_{f}+\alpha_{m})$	$\frac{1}{2}(p_{1}-p_{2})(\alpha_{f}+\alpha_{m})$	$p_{1}(\alpha_{f}+\alpha_{m})$
		$\text{mean}=\frac{1}{2}(p_{1}-p_{2})(\alpha_{f}+\alpha_{m})$		
Approach 3b	$-p_{2}(\alpha_{f}+\alpha_{m})$	$\begin{array}{l} a(p_{1}-p_{2}+k_{1}\\ -2p_{1}p_{2}(k_{1}+k_{2})) \end{array}$	$\begin{array}{l} a(p_{1}-p_{2}+k_{2}\\ -2p_{1}p_{2}(k_{1}+k_{2})) \end{array}$	$p_{1}(\alpha_{f}+\alpha_{m})$
		$\text{mean}=\frac{1}{2}(p_{1}-p_{2})(\alpha_{f}+\alpha_{m})$		

**Table 3:** Summary of variance components for all approaches

	Additive variance $\sigma_{A}^{2}$	Dominance variance $\sigma_{D}^{2}$	Covariance between additive and dominance effects $\sigma_{AD}$
Approach 1 and 2b			
Female	$\sigma_{Af}^{2}=2p_{1}p_{2}\alpha_{f}^{2}$	$a^{2}p_{1}p_{2}({\left(k_{1}-k_{2}\right)}^{2}+p_{1}p_{2}{\left(k_{1}+k_{2}\right)}^{2})$	$\sigma_{ADf}=ap_{1}p_{2}\alpha_{f}(k_{2}-k_{1})$
Male	$\sigma_{Am}^{2}=2p_{1}p_{2}\alpha_{m}^{2}$		$\sigma_{ADm}=ap_{1}p_{2}\alpha_{m}(k_{1}-k_{2})$
Approach 2a and 3b	$p_{1}p_{2}(\alpha_{f}^{2}+\alpha_{m}^{2})$	${\left(ap_{1}p_{2}(k_{1}+k_{2})\right)}^{2}$	0
Approach 3a	$\frac{1}{2}p_{1}p_{2}{\left(\alpha_{f}+\alpha_{m}\right)}^{2}$	$\frac{1}{2}a^{2}p_{1}p_{2}({\left(k_{1}-k_{2}\right)}^{2}+2p_{1}p_{2}{\left(k_{1}+k_{2}\right)}^{2})$	0

**Table 4:** Summary of covariances between relatives for all approaches

	Parent-offspring	Full sib	Half sib
Approach 1 and 2b^1^			
Female	$\frac{1}{2}p_{1}p_{2}\alpha_{f}(\alpha_{f}+\alpha_{m})$	$\frac{1}{4}p_{1}p_{2}(2(\alpha_{f}^{2}+\alpha_{m}^{2})+a^{2}p_{1}p_{2}{\left(k_{1}+k_{2}\right)}^{2})$	$\frac{1}{2}p_{1}p_{2}\alpha_{f}^{2}$
Male	$\frac{1}{2}p_{1}p_{2}\alpha_{m}(\alpha_{f}+\alpha_{m})$		$\frac{1}{2}p_{1}p_{2}\alpha_{m}^{2}$
Approach 2a and 3b	$\frac{1}{2}p_{1}p_{2}(\alpha_{f}^{2}+\alpha_{m}^{2})$	$\frac{1}{4}p_{1}p_{2}(\alpha_{f}^{2}+\alpha_{m}^{2})$	$\frac{1}{4}p_{1}p_{2}(2(\alpha_{f}^{2}+\alpha_{m}^{2})+a^{2}p_{1}p_{2}{\left(k_{1}+k_{2}\right)}^{2})$
Approach 3a	$\frac{1}{4}p_{1}p_{2}{\left(\alpha_{f}+\alpha_{m}\right)}^{2}$	$\frac{1}{8}p_{1}p_{2}{\left(\alpha_{f}+\alpha_{m}\right)}^{2}$	$\begin{array}{l} \frac{1}{4}p_{1}p_{2}(\alpha_{f}+\alpha_{m}{)}^{2}+\frac{1}{2}a^{2}p_{1}p_{2}((k_{1}-k_{2}{)}^{2}\\ +2p_{1}p_{2}(k_{1}+k_{2}{)}^{2}) \end{array}$

^1^These covariances between relatives were also derived by Dai and Weeks (2006) using an extension to the Li and Sacks (1954) method of calculating joint genotype probabilities between pairs of relatives. Dai and Weeks (2006) distinguish maternal and paternal genotypes in order to incorporate imprinting.

## Inbreeding and Imprinting

An interesting aspect of the above variance decompositions is the similarity between inbreeding and imprinting, as inbreeding also introduces a covariance between additive and dominance effects (Harris 1964) that may not be partitioned if an incorrect method is used. To investigate this similarity, we incorporate inbreeding into Approach 2b. We represent an inbred population by dividing the population into two groups: a group that represents the expected Hardy-Weinberg proportions, comprising an overall proportion of (1 − *f*), and a completely homozygous group with no heterozygotes, comprising a proportion *f* of the population. Thus genotypic frequencies for *A*_1_*A*_1_, *A*_2_*A*_1_ and *A*_1_*A*_2_, and *A*_2_*A*_2_ genotypes are $p_{1}^{2}+fp_{1}p_{2},$ $p_{1}p_{2}(1-f)$ each and $p_{2}^{2}+fp_{1}p_{2}$, respectively. Now the overall population mean incorporating both inbreeding (*I*) and imprinting is

$$\mu_{\left(I\right)}=ap_{2}(2+p_{1}(1-f)(k_{1}+k_{2})).$$ (5)

When there is no inbreeding, *f* = 0, the population is in Hardy-Weinberg proportions, and the mean reduces to $ap_{2}(2+p_{1}(k_{1}+k_{2}))$ as expected. With no imprinting, the mean reduces to $2ap_{2}(1+kp_{1}(1-f))$. We assume that the inbreeding coefficient *f* is stable across generations, so the proportion of heterozygotes does not change.

As in Approach 2b, male and female additive and dominance deviations may be calculated separately (Santure and Spencer 2006). For example, the additive effect of inheriting an *A*_1_ allele maternally is

$$\begin{array}{c} \varepsilon_{1•(I)}=G_{11}(p_{1}+fp_{2})+G_{12}(p_{2}(1-f))-\mu_{\left(I\right)}\\ =-ap_{2}(1+k_{1}p_{1}-k_{2}p_{2}+f(1-k_{1}p_{1}+k_{2}p_{2})). \end{array}$$

The remaining additive effects are

$$\begin{array}{l} \varepsilon_{•1(I)}=-ap_{2}(1+k_{2}p_{1}-k_{1}p_{2}+f(1-k_{2}p_{1}+k_{1}p_{2}))\\ \varepsilon_{2•(I)}=ap_{1}(1+k_{1}p_{1}-k_{2}p_{2}+f(1-k_{1}p_{1}+k_{2}p_{2}))\\ \varepsilon_{•2(I)}=ap_{1}(1+k_{2}p_{1}-k_{1}p_{2}+f(1-k_{2}p_{1}+k_{1}p_{2})). \end{array}$$

Breeding values and dominance deviations may be calculated as in Approach 2b.

### Genetic variance components

The total variance for an inbred population with imprinting is

$$\sigma_{G(I)}^{2}=\sigma_{G}^{2}+fp_{1}p_{2}(4a^{2}-\alpha_{f}^{2}-\alpha_{m}^{2})-a^{2}f^{2}p_{1}^{2}p_{2}^{2}{\left(k_{1}+k_{2}\right)}^{2}$$

where $\sigma_{G}^{2}$ is the total genetic variance for the case of imprinting only (2). When there is complete inbreeding (*f* = 1), the total variance is

$$\sigma_{G(I,f\to 1)}^{2}=4a^{2}p_{1}p_{2}$$ (6)

and for no inbreeding (*f* = 0), we recover

$$\sigma_{G(I,f\to 0)}^{2}=\sigma_{G}^{2}$$. (7)

The total variance may also be rewritten as

$$\begin{array}{c} \sigma_{G(I)}^{2}=(1-f)\sigma_{G(I,f\to 0)}^{2}+f\sigma_{G(I,f\to 1)}^{2}\\ +\frac{1}{2}f(1-f)(\sigma_{Df(\text{1})}^{2}+\sigma_{Dm(\text{1})}^{2}+2\sigma_{ADf(1)}+2\sigma_{ADm(1)}) \end{array}$$ (8)

and for the case of no imprinting (*k*_1_ = *k*_2_ = *k*), the total genetic variance becomes

$$\sigma_{G(I,\;k_{1}=k_{2}=k)}^{2}=(1-f)\sigma_{G(I,f\to 0,\;k_{1}=k_{2}=k)}^{2}+f\sigma_{G(I,f\to 1)}^{2}+f(1-f)\sigma_{D(k_{1}=k_{2}=k)}^{2}$$ (9)

(Harris, 1964), where

$$\sigma_{G(I,f\to 0,\;k_{1}=k_{2}=k)}^{2}=p_{1}p_{2}(2\alpha^{2}+4a^{2}k^{2}p_{1}p_{2})$$,

$$\alpha =a(1+k(p_{1}-p_{2}))$$ (10)

and

$$\sigma_{D(k_{1}=k_{2}=k)}^{2}=4a^{2}k^{2}p_{1}^{2}p_{2}^{2}$$.

Comparing equations (6) and (7), we can see that in the absence of imprinting and dominance (*k*_1_ = *k*_2_ = *f* = 0), the total variance for an inbred population is twice that of an outbred population (*k*_1_ = *k*_2_ = 0, *f* = 1), and indeed the effect of inbreeding is linear; an inbreeding coefficient of $f=\frac{1}{2}$ yields a total variance $\frac{3}{2}$ times the variance with no inbreeding. However, for dominance but no imprinting (9), the effect of increasing inbreeding is nonlinear; the population variance may increase or decrease relative to an outbred population depending on the allele frequencies and the value of the dominance coefficient. A similar effect is evident with imprinting; if there is no dominance (*i.e.*, *k*_1_ = −*k*_2_), the population variance linearly increases with increasing inbreeding, while with both dominance and imprinting $\left(k_{1}\neq -k_{2}\right)$ the population variance is a quadratic function of *f*. Thus, it is dominance but not imprinting which determines the relationship of the population variance with increasing inbreeding.

For a highly selfing species, the degree of imprinting may have a large effect on the total population variance. For example, consider a population with $f=\frac{1}{2}$ and $a=p_{1}=p_{2}=\frac{1}{2}$. Setting *k*_1_ = −*k*_2_ (imprinting, no dominance), the total variance is 0.20 for $k_{1}=\frac{1}{2}$ and 0.25 for $k_{1}=1$. Interestingly, the effect of imprinting becomes less pronounced as inbreeding levels increase; for $f=\frac{1}{4}$, the total variance increases from 0.18 for $k_{1}=\frac{1}{2}$ to 0.25 for $k_{1}=1$, while for $f=\frac{3}{4}$ the variance increases from 0.23 for $k_{1}=\frac{1}{2}$ to 0.25 for $k_{1}=1$.

The female and male additive variances are

$$\begin{array}{c} \sigma_{Af(I)}^{2}=2p_{1}p_{2}(1+f)(\alpha_{f}+f(2a-\alpha_{f}){)}^{2}\\ =\sigma_{Af}^{2}+2p_{1}p_{2}(f\alpha_{f}^{2}\\ +(1+f)(2f\alpha_{f}(2a-\alpha_{f})+f^{2}(2a-\alpha_{f}{)}^{2})) \end{array}$$ (11)

and

$$\begin{array}{c} \sigma_{Am(I)}^{2}=2p_{1}p_{2}(1+f)(\alpha_{m}+f(2a-\alpha_{m}){)}^{2}\\ =\sigma_{Am}^{2}+2p_{1}p_{2}(f\alpha_{m}^{2}\\ +(1+f)(2f\alpha_{m}(2a-\alpha_{m})+f^{2}(2a-\alpha_{m}{)}^{2})) \end{array}$$ (12)

where $\sigma_{Af}^{2}$ and $\sigma_{Am}^{2}$ are the female and male additive variances calculated from Approaches 1 and 2b (Table 3). The female and male dominance variances are

$$\begin{array}{c} \sigma_{Df(I)}^{2}=\sigma_{D}^{2}+fp_{1}p_{2}(\alpha_{f}^{2}-\alpha_{m}^{2}+4(a-\alpha_{f})(a-\alpha_{m})\\ -f(a^{2}p_{1}p_{2}(k_{1}+k_{2}{)}^{2}+2(2a-\alpha_{f})(2a-2\alpha_{f}-\alpha_{m}))\\ +2f^{2}(2a-\alpha_{f}{)}^{2}) \end{array}$$ (13)

and

$$\begin{array}{c} \sigma_{Dm(I)}^{2}=\sigma_{D}^{2}+fp_{1}p_{2}(\alpha_{m}^{2}-\alpha_{f}^{2}+4(a-\alpha_{f})(a-\alpha_{m})\\ -f(a^{2}p_{1}p_{2}(k_{1}+k_{2}{)}^{2}+2(2a-\alpha_{m})(2a-2\alpha_{m}-\alpha_{f}))\\ +2f^{2}(2a-\alpha_{m}{)}^{2}). \end{array}$$ (14)

where $\sigma_{D}^{2}$ is the dominance variance (Approaches 1 and 2b, Table 3). Interestingly, and unlike the pure imprinting case, the dominance variance is different for males and females. The female and male covariances between additive and dominance terms are

$$\begin{array}{c} \sigma_{ADf(I)}=\sigma_{ADf}-fp_{1}p_{2}(2a(\alpha_{f}-\alpha_{m})+2\alpha_{f}\alpha_{m}\\ +f(2a-\alpha_{f})(3\alpha_{f}+\alpha_{m}+2f(2a-\alpha_{f}))) \end{array}$$ (15)

and

$$\begin{array}{c} \sigma_{ADm(I)}=\sigma_{ADm}-fp_{1}p_{2}(2a(\alpha_{m}-\alpha_{f})+2\alpha_{f}\alpha_{m}\\ +f(2a-\alpha_{m})(3\alpha_{m}+\alpha_{f}+2f(2a-\alpha_{m}))). \end{array}$$ (16)

where $\sigma_{ADf}^{2}$ and $\sigma_{ADm}^{2}$ are the female and male covariances between additive and dominance effects (Approaches 1 and 2b, Table 3). When there is no imprinting, the female and male covariances reduce to the same value:

$$\begin{array}{c} \sigma_{ADf(I,\;k_{1}=k_{2}=k)}=\sigma_{ADm(I,\;k_{1}=k_{2}=k)}\\ =-2a^{2}fp_{1}p_{2}(1+f+k(1-f)(p_{1}-p_{2}){)}^{2}. \end{array}$$ (17)

Considering that $f,p_{1},\;p_{2}\in (0,1)$ we can see that $-2a^{2}fp_{1}p_{2}<0$ and hence the covariance is strictly negative under inbreeding alone. Recall that under imprinting alone

$$\sigma_{ADf}=ap_{1}p_{2}\alpha_{f}(k_{2}-k_{1})$$

and

$$\sigma_{ADm}=ap_{1}p_{2}\alpha_{m}(k_{1}-k_{2})$$

(Table 3). Now we may rearrange $\alpha_{f}$ to give $\alpha_{f}=\alpha_{m}+a(k_{1}-k_{2})$, so that

$$\sigma_{ADf}=-\sigma_{ADm}-a^{2}p_{1}p_{2}{\left(k_{1}-k_{2}\right)}^{2}$$

and hence $\frac{1}{2}(\sigma_{ADf}+\sigma_{ADm})<0$, so the average of male and female covariances under inbreeding is also strictly negative. However, if *k*_1_ and *k*_2_ are of opposite sign, then one of $\sigma_{ADf}$ or $\sigma_{ADm}$ may be positive. Thus, although both imprinting and inbreeding introduce a covariance between additive and dominance effects, it is only the presence of imprinting that allows the covariance in one sex to be positive. Imprinting can therefore have a significant effect on the total genetic variance and on the sex-specific components of variance of an inbred population.

## Response to Selection

We follow the approach of Heywood (2005) to investigate the response of an imprinted quantitative trait to natural selection. To include selection, let the absolute fitness of parent genotype *A_i_A_j_* be $\varphi_{ij},$ and define the relative fitness *w_ij_* as $\varphi_{ij}/\overset{-}{\varphi }$ where

$$\overset{-}{\varphi }=\sum_{i,j=1}^{2}p_{i}p_{j}\varphi_{ij}$$ (18)

is the mean fitness. Following Heywood (2005), we consider the special case with the linear fitness function $\varphi_{ij}=1+G_{ij}t$, which gives $w_{ij}=(1+G_{ij}t)/\overset{-}{\varphi }$. We denote mean offspring genotypic values after selection as $G_{ij}^{\prime }$. We can now write the change in mean trait value from the parent to the offspring generation (the response to selection; Δμ) as

$$\begin{array}{c} \Delta \mu \;=\sigma_{Gw}+E(w\Delta G)\\ =S+E(w\Delta G) \end{array}$$ (19)

where $\sigma_{Gw}=S$, the selection differential, is the covariance between parent relative fitness and genotypic value, $\Delta G=G^{\prime }-G$ is the change in mean trait value from parent to offspring, and the expectation is taken over parents (Heywood 2005; Price 1970; Price 1972).

Heywood (2005) defines $G_{ij}^{*}$ as the mean genotypic value of offspring from parent *A_i_A_j_* before selection, then sets $\delta =G^{\prime }-G^{*}$ and, after some algebra, restates (19) as

$$\Delta \mu \;=\beta_{G^{*}G}S+\sigma_{wG^{*}•G}+\sigma_{w\delta }+E(\delta )+E(G^{*}-G)$$ (20)

or, alternatively,

$$\Delta \mu \;=\beta_{G^{\prime }G}S+\sigma_{wG^{*}•G}+\sigma_{w\delta •G}+E(\delta )+E(G^{*}-G)$$ (21)

(Heywood 2005). We now apply this approach to an imprinted quantitative trait. As usual, we need to define both male (paternal) and female (maternal) terms. The absolute fitnesses of the four genotypes are

$$\begin{array}{c} \varphi_{11}=1\\ \varphi_{21}=1+at(1+k_{1})\\ \varphi_{12}=1+at(1+k_{2})\\ \varphi_{22}=1+2at \end{array}$$

and

$$\begin{array}{c} \overset{-}{\varphi }=\sum_{i,j=1}^{2}p_{i}p_{j}\varphi_{ij}\\ =1+t\mu . \end{array}$$ (22)

Relative fitnesses are shown in Table 5, along with the frequency of each genotype and average values of offspring before and after selection. Note that the population mean, variances and covariances $\left(\mu ,\;\sigma_{G}^{2},\;\sigma_{Af}^{2},\;\sigma_{Am}^{2},\;\sigma_{D}^{2},\;\sigma_{ADf}\text{ and }\sigma_{ADm}\right)$ are the same as derived for Approaches 1 and 2b (Table 3).

**Table 5:** Population values under selection model

Genotype	$A_{1}A_{1}$	$A_{2}A_{1}$	$A_{1}A_{2}$	$A_{2}A_{2}$
Genotypic value	0	$a(1+k_{1})$	$a(1+k_{2})$	2a
Frequency before selection	$p_{1}^{2}$	$p_{2}p_{1}$	$p_{1}p_{2}$	$p_{2}^{2}$
Fitness	$1/\overset{-}{\varphi }$	$(1+at(1+k_{1}))/\overset{-}{\varphi }$	$(1+at(1+k_{2}))/\overset{-}{\varphi }$	$(1+2at)/\overset{-}{\varphi }$
Average value of offspring before selection: maternal	$ap_{2}(1+k_{2})$	$\frac{1}{2}a(p_{1}(1+k_{1})+p_{2}(3+k_{2}))$	$\frac{1}{2}a(p_{1}(1+k_{1})+p_{2}(3+k_{2}))$	$a(p_{1}(1+k_{1})+2p_{2})$
Average value of offspring before selection: paternal	$ap_{2}(1+k_{1})$	$\frac{1}{2}a(p_{1}(1+k_{2})+p_{2}(3+k_{1}))$	$\frac{1}{2}a(p_{1}(1+k_{2})+p_{2}(3+k_{1}))$	$a(p_{1}(1+k_{2})+2p_{2})$
Frequency after selection	$p_{1}^{2}/\overset{-}{\varphi }$	$p_{2}p_{1}(1+at(1+k_{1}))/\overset{-}{\varphi }$	$p_{1}p_{2}(1+at(1+k_{2}))/\overset{-}{\varphi }$	$p_{2}^{2}(1+2at)/\overset{-}{\varphi }$
Average value of offspring after selection: maternal	$ap_{2}^{\prime }(1+k_{2})$	$\frac{1}{2}a(p_{1}^{\prime }(1+k_{1})+p_{2}^{\prime }(3+k_{2}))$	$\frac{1}{2}a(p_{1}^{\prime }(1+k_{1})+p_{2}^{\prime }(3+k_{2}))$	$a(p_{1}^{\prime }(1+k_{1})+2p_{2}^{\prime })$
Average value of offspring after selection: paternal	$ap_{2}^{\prime }(1+k_{1})$	$\frac{1}{2}a(p_{1}^{\prime }(1+k_{2})+p_{2}^{\prime }(3+k_{1}))$	$\frac{1}{2}a(p_{1}^{\prime }(1+k_{2})+p_{2}^{\prime }(3+k_{1}))$	$a(p_{1}^{\prime }(1+k_{2})+2p_{2}^{\prime })$

Now the allele frequencies after selection are

$$p_{1}^{\prime }=\frac{p_{1}(2+ap_{2}t(2+k_{1}+k_{2}))}{2(1+ap_{2}t(2+p_{1}(k_{1}+k_{2})))}$$ (23)

and

$$p_{2}^{\prime }=\frac{p_{2}(2+at(4+p_{1}(-2+k_{1}+k_{2}))}{2(1+ap_{2}t(2+p_{1}(k_{1}+k_{2})))}$$ (24)

For both female and male parents, the mean genotypic value of offspring before selection is equal to the mean genotypic value:

$${\overset{-}{G}}_{f}^{*}=\sum_{i,j=1}^{2}p_{i}p_{j}G_{ijf}^{*}=\mu$$ (25)

$${\overset{-}{G}}_{m}^{*}=\sum_{i,j=1}^{2}p_{i}p_{j}G_{ijm}^{*}=\mu$$ (26)

The mean values of offspring after selection for female and male parents are:

$$\begin{array}{l} {\overset{-}{G}}_{f}^{\prime }=\sum_{i,j=1}^{2}p_{i}p_{j}G_{ijf}^{\prime }\\ {\overset{-}{G}}_{m}^{\prime }=\sum_{i,j=1}^{2}p_{i}p_{j}G_{ijm}^{\prime } \end{array}$$

The difference between male and female offspring means after selection is

$${\overset{-}{G}}_{f}^{\prime }-{\overset{-}{G}}_{m}^{\prime }=\frac{1}{2}ap_{1}p_{2}t(k_{2}-k_{1})(\alpha_{f}+\alpha_{m})/\overset{-}{\varphi }$$ (27)

which is zero when there is no imprinting (*k*_1_ = *k*_2_ = *k*). This result clearly demonstrates the difference between female and male parents in their effect on offspring means.

We derive the full set of covariances and expectations required for equations (20) and (21) in the Appendix. Now, the response to selection is

$$\begin{array}{c} \Delta \mu_{f}=t\gamma (\overset{-}{\varphi }-\frac{1}{2}t\psi )/{\overset{-}{\varphi }}^{2}\\ \Delta \mu_{m}=\Delta \mu_{f} \end{array}$$ (28)

where

$$\psi =ap_{1}p_{2}(k_{1}+k_{2})=\sqrt{\sigma_{D}^{2}+\sigma_{ADf}+\sigma_{ADm}}$$ (29)

and

$$\gamma =\frac{1}{2}(\sigma_{Af}^{2}+\sigma_{ADf}+\sigma_{Am}^{2}+\sigma_{ADm}).$$ (30)

It is clear, therefore, that the response to selection is the same for males and females, and is, as expected, related to the population variances and covariances in addition to the selection coefficient *t*. In the absence of imprinting *k*_1_ = *k*_2_ = *k*, $\gamma =2p_{1}p_{2}\alpha^{2}$, where $\alpha =a(1+k(p_{1}-p_{2}))$, and $\psi =2akp_{1}p_{2}$, and our total response to selection becomes

$$\Delta \mu =t\sigma_{A}^{2}({\overset{-}{\varphi }}_{\left(k_{1}=k_{2}=k\right)}-\frac{1}{2}t\sigma_{D})/{\overset{-}{\varphi }}_{\left(k_{1}=k_{2}=k\right)}^{2}$$ (31)

(Heywood 2005) where

$$\sigma_{A}^{2}=2p_{1}p_{2}\alpha^{2},$$

$$\sigma_{D}^{2}={\left(2akp_{1}p_{2}\right)}^{2}$$

and

$${\overset{-}{\varphi }}_{\left(k_{1}=k_{2}=k\right)}=1+2ap_{2}t(1+kp_{1}).$$

How does the magnitude of the response to selection compare to what we would predict if imprinting is ignored, and reciprocal heterozygotes are assumed to have the same genotypic value? Substituting $k=\frac{1}{2}(k_{1}+k_{2})$ into (31), we find that the expression for the response to selection is identical to the full expression derived with separate *k*_1_ and *k*_2_ terms (28). This suggests that even if imprinting is acting, the predicted response to selection is the same whether calculated using separate genotypic values, or using the average of the genotypic values for the two reciprocal heterozygotes. If *k*_1_ = −*k*_2_ so there is imprinting but no dominance (as the mean heterozygote genotypic value is midway between the homozygote genotypic values; $k=\frac{1}{2}(k_{1}+k_{2})=0$), expressions (28) and (31) become

$$\Delta \mu =2a^{2}p_{1}p_{2}t/(1+2ap_{2}t).$$ (32)

### Comparison to breeder’s equation

The response to selection according to the breeder’s equation is

$$\Delta \mu \;=h^{2}S$$ (33)

where the narrow sense heritability, *h*^2^, is the ratio between the additive and total genetic variance and $S=\sigma_{Gw}=t\sigma_{G}^{2}/\overset{-}{\varphi }$ as previously. For the case of imprinting, we can see that the breeder’s equation

$$\begin{array}{c} \Delta \mu =\sigma_{A(1)}^{2}/\sigma_{G}^{2}•\sigma_{wG}\\ =\sigma_{A(1)}^{2}/\sigma_{G}^{2}•t\sigma_{G}^{2}/\overset{-}{\varphi }\\ =t\sigma_{A(1)}^{2}/\overset{-}{\varphi } \end{array}$$ (34)

is only equal to the response to selection

$$\begin{array}{c} \Delta \mu =t\gamma (\overset{-}{\varphi }-\frac{1}{2}t\psi )/{\overset{-}{\varphi }}^{2} \end{array}$$ (28)

when the dominance variance and the male and female covariances between the additive and dominance terms are zero, which requires *k*_1_ = *k*_2_ = 0. For the case of no imprinting, the breeder’s equation becomes

$$\Delta \mu =t\sigma_{A}^{2}/{\overset{-}{\varphi }}_{\left(k_{1}=k_{2}=k\right)}$$ (35)

and is equal to the response to selection (31) when $\sigma_{D}=0$. Therefore, we can see the well-known result that the response to selection and the breeder’s equation are equal only when the dominance variance is zero, and hence the breeder’s equation only predicts the response to selection in the absence of dominance, whether the locus is imprinted or not.

The difference between the breeder’s equation (34) and the predicted response to selection (28) is a function dependent on *a*, *t*, *k*_1_, *k*_2_ and *p*_1_ (= 1 − *p*_2_). For a dominant trait with no imprinting $\left(k_{1}=k_{2}=k\neq 0\right)$ the true response to selection (31) is strictly less than that predicted by the breeder’s equation (35). Similarly, if *k*_1_ = −*k*_2_ so there is imprinting but no dominance (as the mean heterozygote genotypic value is midway between the homozygote genotypic values), the breeder’s equation becomes

$$\begin{array}{c} \Delta \mu =t\sigma_{A(1,k_{1}=-k_{2})}^{2}/{\overset{-}{\varphi }}_{\left(k_{1}=-k_{2}\right)}\\ =2a^{2}p_{1}p_{2}t(1+k_{1}^{2})/(1+2ap_{2}t) \end{array}$$ (36)

while the true response to selection is

$$\Delta \mu =2a^{2}p_{1}p_{2}t/(1+2ap_{2}t).$$ (37)

Comparing equations (36) and (37), we can see that the breeder’s equation again overestimates the response to selection for the special case of imprinting but no dominance. For the case of complete inactivation of the maternal or paternal allele $\left(k_{1}^{2}=1\right)$, the breeder’s equation predicts a response double that of the true response.

If we include both imprinting and dominance, and let $a=\frac{1}{2}$, *t* = 1, $k_{1}\in (-1,0)$, $k_{2}\in (0,1)$ and $p_{1}\in (0,1)$, the response to selection (28) is also generally less than that predicted by the breeder’s equation. However, it is interesting to note that if the difference between *k*_1_ and *k*_2_ is less than ≈0.1, then the predicted response to selection may be the same as or slightly more than that predicted by the breeder’s equation. Therefore, very small differences in the genotypic values of reciprocal heterozygotes may result in the breeder’s equation underestimating the response to selection.

These results contrast with the derivation of de Vries *et al.* (1994), who from the covariance of parents and offspring predicted the response to selection for an imprinted trait as

$$\Delta \mu =S(h^{2}+\frac{1}{2}\sigma_{p}^{2})$$ (38)

where $\sigma_{p}^{2}$ is defined as the variance due to imprinted genes.

## Discussion

We have demonstrated that a simple one-locus two-allele model of genomic imprinting produces large differences in predictions for additive (Table 2) and dominance terms from a number of standard approaches for partitioning the genotypic value of an individual. These approaches are equivalent in the absence of imprinting under standard Mendelian expression (where heterozygotes have equivalent genotypic values and hence *k*_1_ = *k*_2_). Although all approaches give identical total genetic variance, there are differences in the partitioning of the genetic variance into additive, dominance and covariance terms (Table 3).

The major differences in the approaches arise due to differences in how breeding values and additive effects are defined. Approaches 1 and 2b incorporate both sex- and generation-dependent terms, and breeding values are equivalent for these approaches (Table 2). However, Approaches 2a and the regression methods (Approaches 3a and 3b) are unable to partition separate male and female terms. Consider how breeding values are calculated for the different approaches. Approach 1 defines breeding values in terms of allelic contribution to offspring, and breeding values are the same for reciprocal heterozygotes. Genotypic values in Approach 2b are defined in terms of the male or female effect they pass on to offspring, and so include the same sex-specific generation effect as Approach 1. Breeding values are consequently equivalent for reciprocal heterozygotes. The single regression Approach 3a similarly forces genotypic equivalence for the predicted value of reciprocal heterozygotes. In contrast, the other two approaches define breeding values in terms of an individual’s own genotype and the parental origin of alleles in that genotype. As a consequence of imprinting, the parental origin of these alleles has an effect on the genotypic value of individuals and hence reciprocal heterozygotes have different breeding values (Table 2).

Under standard Mendelian expression, breeding values are expected to be equivalent whether defined as the sum of additive allelic effects (Approaches 2 and 3) or from the means of offspring (Approach 1). However, differences have been noted where alleles in the population are not in Hardy-Weinberg equilibrium (Ewens 1979), in relation to populations with nonrandom mating and inbreeding (Falconer 1985; Fisher 1941; Templeton 1987), and as a result of population subdivision (Goodnight 2000). Genomic imprinting represents a distinct phenomenon causing differences in the definition of additive effects between the approaches we have investigated. The difference of these approaches in predicted breeding values mirrors the conclusion of Falconer (1985), who found that, “the concept of breeding value [has] no useful meaning when mating is not random.” In addition, genomic imprinting introduces a covariance between breeding values and dominance deviations (Spencer 2002). This covariance between additive and dominance effects has only been noted previously when a population is inbred (Harris 1964).

In comparing these approaches, we assumed that Approach 1 gives us “correct” values for population parameters. Approach 1 is the most time-intensive method for partitioning genetic variance because it requires derivation of mating tables to give offspring mean values. However, this approach does allow separate calculation of male and female variances and covariances, which is of great value when considering offspring-parent and halfsib covariances in real populations.

Approach 2a was able to retrieve the additive variance, but the true additive-by-dominance covariance was included in the expression for the dominance variance. By defining additive terms specific to male and female inheritance, we were able to “rescue” this method to include separate breeding values and dominance deviations, and their corresponding variances, for the two sexes (Approach 2b). Of particular note is that Approach 2b was the only approach able to recover the Approach 1 covariance between additive and dominance effects. Defining separate male and female dominance terms $(\lambda_{ijf}=G_{ij}-\mu -\varepsilon_{i•}-\varepsilon_{j•}$ and $\lambda_{ijm}=G_{ij}-\mu -\varepsilon_{•i}-\varepsilon_{•j})$ includes a “generation” effect that is not accounted for in Approaches 2a and 3. Approach 1 is based on calculating breeding values and dominance deviations that relate to the following generation because we use offspring means in their calculation. The equivalence of Approach 1 with Approach 2b is a reassurance that defining separate male and female dominance terms is an appropriate measure to include a sex and generation effect in this approach. A closer investigation of Approaches 1 and 2b is presented for a model including maternal genetic effects and genomic imprinting (Santure and Spencer 2006).

It is well known that parental effects may have a significant effect on the phenotype of offspring. It is important for methods to include such effects, but it is not easy to imagine how the linear regression models (Approaches 3a and 3b) could be extended to allow for parental effects such as imprinting and maternal genetic effects.

It is interesting to assess how different these approaches are in their estimation of variance and covariance components. The numerical examples in Table 6 contrast genetic variance components and resemblances between relatives for the different approaches for two scenarios, one where alleles are largely paternally inactivated, and one where maternally inherited alleles are largely inactivated. We assume that phenotypic (and hence genotypic) values range from 0 to 1 $\left(a=\frac{1}{2}\right)$. We can see that, as one would expect, paternal inactivation increases the covariance between mothers and offspring and half sibs sharing a mother, relative to fathers and offspring and half sibs sharing a father respectively (and vice versa) (from the correct expressions using Approaches 1 and 2b). Approach 3a underestimates the true additive variance, while Approaches 2a, 3a, and 3b all underestimate the dominance variance. As discussed previously, Approaches 2a, 3a, and 3b are not able to calculate the covariance between additive and dominance effects (Table 6). This covariance between breeding values and dominance deviations is included in the expressions for resemblance between parents and offspring and full sibs and is likely to play a large role in identifying quantitative traits that are influenced by imprinted loci (Spencer 2002).

**Table 6:** Values of variances and covariances for all approaches, given paternal and maternal inactivation

	Paternal inactivation $p_{1}=\frac{1}{2},p_{2}=\frac{1}{2},a=\frac{1}{2},k_{1}=\frac{9}{10},k_{2}=-\frac{8}{10}$			Maternal inactivation $p_{1}=\frac{1}{3},p_{2}=\frac{2}{3},a=\frac{1}{2},k_{1}=-\frac{7}{10},k_{2}=\frac{95}{100}$		
	Approaches 1 and 2b	Approaches 2a and 3b	Approach 3a	Approaches 1 and 2b	Approaches 2a and 3b	Approach 3a
Additive variance						
Female	0.4278	0.2153	0.1250	0.0020	0.1777	0.1020
Male	0.0028			0.3534		
Dominance variance	0.1808	0.0002	0.0905	0.1520	0.0008	0.0764
Additive by dominance covariance						
Female	−0.1966	0	0	0.0122	0	0
Male	0.0159			−0.1635		
Offspring-parent covariance						
Female	0.1156	0.1077	0.0625	0.0071	0.0888	0.0510
Male	0.0094			0.0949		
Half-sib covariance						
Female	0.1070	0.0538	0.0313	0.0005	0.0444	0.0255
Male	0.0007			0.0883		
Full-sib covariance	0.1077	0.1077	0.0851	0.0890	0.0890	0.0701

By using Approach 2b, we were able to extend the imprinting model to include inbreeding. As previously noted, inbreeding also creates differences in how breeding values are defined (see Falconer 1985) and creates a covariance between additive and dominance effects that is not present in a randomly mating population (Harris 1964). Interestingly, we have demonstrated that in the presence of both inbreeding and imprinting, the dominance variance is different for males and females. The covariance between additive and dominance terms is strictly negative under inbreeding alone, and is on average negative when averaged over males and females under imprinting alone. However, it is only imprinting that allows the covariance in one sex to be positive. The sex-based differences introduced by imprinting represent an important difference between the effects of inbreeding and imprinting on the derivation of quantitative genetic models.

Finally, we derived the full expression for the response to selection of an imprinted trait. For an imprinted trait, the breeder’s equation generally overestimates the true response to selection, a result well established when a trait is known to exhibit dominance. Excitingly, we have demonstrated that even in the absence of dominance, where on average reciprocal heterozygotes have a genotypic value midway between the two homozygotes, the breeder’s equation does not predict the true response to selection. This result has very great significance for predicting the reaction to selection in natural populations—if heterozygotes are not distinguished and we only measure additive variance, we are very likely to overestimate the expected change in mean trait values between generations.

Detecting genomic imprinting of a quantitative trait using, for example, covariances between relatives, is likely to be difficult given the large sampling variance of such covariances and the possibility of maternal effects increasing the covariance of offspring with their mothers (Santure and Spencer 2006; Spencer 2002). However, the derivations above do suggest that a number of different quantities may provide indicators for the influence of imprinting, such that if one approach lacks power to distinguish imprinting from nonimprinting, another avenue may provide fruitful. For example, 1) large differences in the covariance of offspring with their mothers compared to fathers (particularly if the covariance with fathers is greater), 2) the existence of a non-zero covariance between additive effects and dominance deviations (particularly if there is a difference in sign between male and female covariances), and 3) a smaller than expected response to selection based on the breeder’s equation (particularly when there is little evidence for dominance) all provide good evidence for the influence of genomic imprinting on a quantitative trait. A large range of methods is presently available for assessing the role of imprinting in complex and quantitative traits. These methods follow the broad spectrum of genetic approaches for dissecting complex traits, from general mixed models, use of covariances between relatives and identification of parent of origin effects in phenotype inheritance for traits without genotypic information available; to the marker-based approaches of linkage mapping, association studies and QTL mapping. A number of these approaches utilize variance component estimation, resemblances between relatives or differences in the phenotypic values of heterozygotes; quantities discussed in this manuscript. Such approaches are invaluable in the dissection of quantitative traits, and we encourage researchers to employ an approach that can successfully incorporate genomic imprinting into a model of the quantitative trait of interest.

## Appendix

### Approach 1 (Falconer and Mackay 1996)

This approach is based on using the genotypic values of parents and offspring to calculate genotypic deviations, population breeding values and dominance deviations, components of variance, and covariances between relatives. The genotypic deviation of a genotype is the difference between its genotypic value (*G_ij_*) and the population mean $\left(\mu =ap_{2}(2+p_{1}(k_{1}+k_{2}))\right)$. The breeding value is defined as twice the difference between the mean genotypic value of the class’s offspring and the population mean, and can be derived separately for males and females (Spencer 2002). The dominance deviation for a genotypic class is the difference between the genotypic deviation and the breeding value. The genetic variance of the population is the variance of the genotypic deviations, male and female additive genetic variances are the respective variances of their breeding values and the male and female dominance genetic variances are the variances of the male and female dominance deviations (Spencer 2002). Resemblances between relatives are calculated from first principles with the help of a mating table.

### Approach 2a (Lynch and Walsh 1998)

Based on a general least squares approach to calculate population breeding values, dominance deviations, components of variance and covariances between relatives. The genotypic value *G_ij_* for genotype *A_i_A_j_* is the sum of the population mean (μ) plus additive (ε) and dominance (λ) effects:

$$G_{ij}=\mu +\varepsilon_{i•}+\varepsilon_{•j}+\lambda_{ij}$$

where $\varepsilon_{i•}$ is the average additive effect of inheriting an *A_i_* allele from the mother, $\varepsilon_{•j}$ is the average effect of inheriting an *A_j_* allele from the father and $\lambda_{ij}$ is the remaining dominance term (also see Santure and Spencer 2006). Note that here “•” represents either of an *A*_1_ or *A*_2_ allele in the genotype. Breeding values are defined as the sum of additive effects of alleles for each genotype, for example the breeding value of the *A*_1_*A*_2_ genotype is $\varepsilon_{1•}+\varepsilon_{•2}$. The additive variance is the variance of the additive allelic effects, while the dominance variance is the variance of the dominance deviations. By definition, the covariance between additive allelic and dominance effects is zero.

In the absence of separate female and male variances, we follow Fisher (1918) and define the covariances between relatives as sums of additive and dominance variances.

### Approach 2b (Lynch and Walsh 1998)

Approach 2a (above, and in Santure and Spencer 2006) calculated total additive and dominance effects and did not allow separate calculation of female and male additive and dominance variances as was possible in Approach 1. By treating individuals as parents in terms of the alleles that they will pass onto offspring in the next generation, we can redefine the genotypic value of an individual as the sum of additive effects inherited by its offspring, plus the population mean and a sex-specific dominance deviation (Santure and Spencer 2006). In using these definitions, we partition the additive and dominance terms into those specific to male and female inheritance.

Now the partitioning of the genotypic value becomes different for males and females:

$$\begin{array}{c} G_{ij}=\mu +\varepsilon_{i•}+\varepsilon_{j•}+\lambda_{ijf}\\ =\mu +\varepsilon_{•i}+\varepsilon_{•j}+\lambda_{ijm} \end{array}$$

where the extra subscript on λ indicates female (*f*) and male (*m*) dominance effects, defined as

$$\lambda_{ijf}=G_{ij}-\mu -\varepsilon_{i•}-\varepsilon_{j•}$$

and

$$\lambda_{ijm}=G_{ij}-\mu -\varepsilon_{•i}-\varepsilon_{•j}$$

Male and female breeding values are then defined as the sum of male and female additive effects;

$$\begin{array}{c} {\text{bv}}_{f}(A_{i}A_{j})=\varepsilon_{i•}+\varepsilon_{j•}\\ {\text{bv}}_{m}(A_{i}A_{j})=\varepsilon_{•i}+\varepsilon_{•j} \end{array}$$

The male and female additive genetic variances are the variances of male and female additive effects, dominance genetic variances are the variances of the dominance deviations, and the covariance between dominance deviations and breeding values is similarly calculated separately for males and females. Covariances between relatives are then calculated following Spencer (2002) as sums of additive, dominance and covariance terms.

### Approach 3a (Fisher 1918; Lynch and Walsh 1998)

An alternative approach is to follow a regression method, expressing the genotypic value *G_ij_* of the *A_i_A_j_* genotype using least squares regression (Fisher 1918): based on the relationship between the number of copies of the *A*_2_ allele in the genotype and the genotypic value, we may define *G_ij_* as the sum of a predicted regression value $\left({\hat{G}}_{ij}\right)$ and a residual error corresponding to a dominance deviation $\left(\lambda_{ij}\right)$. The predicted regression value may be further decomposed into the mean of the genotypes (μ) plus additive effects $\left(\varepsilon_{i}\right)$, where additive effects are linear terms dependent on the number of *A*_1_ and *A*_2_ alleles in the genotype (*N*_1_ and *N*_2_ = (2 − *N*_1_) respectively), so that

$$G_{ij}={\hat{G}}_{ij}+\lambda_{ij}=\mu +N_{1}\varepsilon_{1}+N_{2}\varepsilon_{2}+\lambda_{ij}$$

Breeding values, dominance terms, variances, and covariances are calculated as in Approach 2a. By definition, the covariance between additive and dominance terms is zero.

### Approach 3b (Lynch and Walsh 1998)

Alternatively, we may extend to a multiple regression approach to dissect the genotypic value into additive and dominance components. Using matrix notation, we can express the genotypic value as

$$G_{ij}=\text{X}\beta +\lambda$$

where *G_ij_* is the matrix of genotypic values, X is an incidence matrix, β is the vector of the intercept (κ) and the two parental partial regression coefficients $(\tau_{female}$ and $\tau_{male})$and δ is the vector of dominance effects:

$$G_{ij}=\left[\begin{array}{c} G_{11}\\ G_{21}\\ G_{12}\\ G_{22} \end{array}\right]=\left[\begin{array}{c} 0\\ a(1+k_{1})\\ a(1+k_{2})\\ 2a \end{array}\right],$$

$$\text{X}=\left[\begin{array}{ccc} 1 & 0 & 0\\ 1 & 1 & 0\\ 1 & 0 & 1\\ 1 & 1 & 1 \end{array}\right],\beta =\left[\begin{array}{c} \kappa \\ \tau_{female}\\ \tau_{male} \end{array}\right],$$

$$\lambda =\left[\begin{array}{c} \lambda_{11}\\ \lambda_{21}\\ \lambda_{12}\\ \lambda_{22} \end{array}\right]$$

The terms $\kappa \text{, }\tau_{female}$ and $\tau_{male}$ may then be estimated using a generalized least squares approach, so that

$$\hat{\beta }={\left(X^{T}\text{RX}\right)}^{-1}X^{T}\text{R}G_{ij}$$

where

$$\text{R}=\text{diag}(\begin{array}{cccc} p_{1}^{2} & p_{2}p_{1} & p_{1}p_{2} & p_{2}^{2} \end{array})$$

is the matrix of genotypic frequencies. Additive effects for each genotype are defined as the difference between the genotypic value and the sum of the population mean and dominance effect. Breeding values, dominance terms, variances and covariances are calculated as in Approach 2a. By definition, the covariance between additive and dominance terms is zero.

### Response to selection

We here derive the full set of covariances and expectations which, in addition to equations (22)–(26), are required to describe the response to selection of an imprinted trait [equations (20) and (21)].

The covariances between genotypic values before and after selection are

$$\begin{array}{c} \sigma_{GG_{f}^{\prime }}=\sum_{i,j=1}^{2}p_{i}p_{j}G_{ij}G_{ijf}^{\prime }-\mu {\overset{-}{G}}_{f}^{\prime }\\ =\frac{1}{4}(\sigma_{Af}^{2}+2p_{1}p_{2}\alpha_{f}\alpha_{m}+p_{1}p_{2}t(\alpha_{f}+\alpha_{m})(4ap_{2}\alpha_{f}+a^{2}p_{1}p_{2}(k_{1}^{2}-k_{2}^{2})))/\overset{-}{\varphi } \end{array}$$

and

$$\sigma_{GG_{m}^{\prime }}=\frac{1}{4}(\sigma_{Am}^{2}+2p_{1}p_{2}\alpha_{f}\alpha_{m}+p_{1}p_{2}t(\alpha_{f}+\alpha_{m})(4ap_{2}\alpha_{m}+a^{2}p_{1}p_{2}(k_{2}^{2}-k_{1}^{2})))/\overset{-}{\varphi }.$$

The covariance between selection coefficients and genotypic values after selection are

$$\begin{array}{c} \sigma_{G_{f}^{\prime }w}=\sum_{i,j=1}^{2}p_{i}p_{j}G_{ijf}^{\prime }w_{ij}-{\overset{-}{G}}_{f}^{\prime }\\ =t\sigma_{GG_{f}^{\prime }}/\overset{-}{\varphi }\\ \sigma_{G_{m}^{\prime }w}=t\sigma_{GG_{m}^{\prime }}/\overset{-}{\varphi }. \end{array}$$

Thus, although the mean values of offspring after selection for female and male parents differ, the relationship between selection coefficients and the difference in genotypic values before and after selection are the same for the offspring of female and male parents. Other covariances are shown below

$$\begin{array}{c} \sigma_{Gw}=\sum_{i,j=1}^{2}p_{i}p_{j}G_{ij}w_{ij}-\mu \\ =t\sigma_{G}^{2}/\overset{-}{\varphi } \end{array}$$

$$\begin{array}{c} \sigma_{GG_{f}^{*}}=\sum_{i,j=1}^{2}p_{i}p_{j}G_{ij}G_{ijf}^{*}-\mu^{2}\\ =\frac{1}{2}(\sigma_{Af}^{2}+\sigma_{ADf})\\ \sigma_{GG_{m}^{*}}=\frac{1}{2}(\sigma_{Am}^{2}+\sigma_{ADm}) \end{array}$$

$$\begin{array}{c} \sigma_{G_{f}^{*}w}=\sum_{i,j=1}^{2}p_{i}p_{j}G_{ijf}^{*}w_{ij}-\mu \\ =t\sigma_{GG_{f}^{*}}/\overset{-}{\varphi }\\ \sigma_{G_{m}^{*}w}=t\sigma_{GG_{m}^{*}}/\overset{-}{\varphi } \end{array}$$

$$\begin{array}{c} \sigma_{w\delta_{f}}=\sum_{i,j=1}^{2}p_{i}p_{j}w_{ij}\delta_{ijf}-E(\delta_{f})\\ =-\frac{1}{4}t^{2}(ap_{1}^{2}p_{2}^{2}(k_{1}+k_{2})(\alpha_{f}+\alpha_{m}{)}^{2})/{\overset{-}{\varphi }}^{2}\\ =\sigma_{w\delta m} \end{array}$$

$$\begin{array}{c} E(\delta_{f})=\sum_{i,j=1}^{2}p_{i}p_{j}\delta_{ijf}\\ =\frac{1}{4}t(\sigma_{Af}^{2}+\sigma_{Am}^{2}+2\sigma_{ADf})/\overset{-}{\varphi }\\ E(\delta_{m})=\frac{1}{4}t(\sigma_{Af}^{2}+\sigma_{Am}^{2}+2\sigma_{ADm})/\overset{-}{\varphi }=E(\delta_{f}). \end{array}$$

Now we can find the components of the response to selection. Recalling equations (20) and (21), the components of equation (20) for females are

$$\begin{array}{c} \beta_{G_{f}^{*}G}S=(\sigma_{GG_{f}^{*}}/\sigma_{G}^{2})S\\ =\sigma_{GG_{f}^{*}}\sigma_{Gw}/\sigma_{G}^{2}\\ =\frac{1}{2}t(\sigma_{Af}^{2}+\sigma_{ADf})/\overset{-}{\varphi } \end{array}$$ (39)

$$\begin{array}{c} \sigma_{G_{f}^{*}w•G}=\sigma_{G_{f}^{*}w}-\sigma_{Gw}\sigma_{GG_{f}^{*}}/\sigma_{G}^{2}\\ =0 \end{array}$$ (40)

$$\sigma_{w\delta_{f}}=-\frac{1}{4}t^{2}(ap_{1}^{2}p_{2}^{2}(k_{1}+k_{2}){\left(\alpha_{f}+\alpha_{m}\right)}^{2})/{\overset{-}{\varphi }}^{2}$$ (41)

$$E(\delta_{f})=\frac{1}{4}t(\sigma_{Af}^{2}+\sigma_{Am}^{2}+2\sigma_{ADf})/\overset{-}{\varphi }$$ (42)

$$\begin{array}{c} E(G_{f}^{*}-G)=\sum_{i,j=1}^{2}p_{i}p_{j}(G_{ijf}^{*}-G_{ij})\\ =0 \end{array}$$ (43)

and similarly, the components of (20) for males are

$$\beta_{G_{m}^{*}G}S=\frac{1}{2}t(\sigma_{Am}^{2}+\sigma_{ADm})/\overset{-}{\varphi }$$ (44)

$$\sigma_{G_{m}^{*}w•G}=0$$ (45)

$$\sigma_{w\delta_{m}}=-\frac{1}{4}t^{2}(ap_{1}^{2}p_{2}^{2}(k_{1}+k_{2}){\left(\alpha_{f}+\alpha_{m}\right)}^{2})/{\overset{-}{\varphi }}^{2}$$ (46)

$$E(\delta_{m})=\frac{1}{4}t(\sigma_{Am}^{2}+\sigma_{Af}^{2}+2\sigma_{ADm})/\overset{-}{\varphi }$$ (47)

$$E(G_{m}^{*}-G)=0.$$ (48)

Interestingly, $\sigma_{w\delta_{f}}=\sigma_{w\delta_{m}}=\sigma_{w\delta }$ —the covariance between selection coefficients and the change in mean genetic value before and after selection—is the same for offspring of male and female parents. Then we find that the male and female sum of equation (20) components are

$$\begin{array}{c} \Delta \mu_{f}=t\gamma (\overset{-}{\varphi }-\frac{1}{2}t\psi )/{\overset{-}{\varphi }}^{2}\\ \Delta \mu_{m}=\Delta \mu_{f} \end{array}$$ (49)

where

$$\psi =ap_{1}p_{2}(k_{1}+k_{2})=\sqrt{\sigma_{D}^{2}+\sigma_{ADf}+\sigma_{ADm}}$$ (50)

and

$$\gamma =\frac{1}{2}(\sigma_{Af}^{2}+\sigma_{ADf}+\sigma_{Am}^{2}+\sigma_{ADm}).$$ (51)

Hence

$$\Delta \mu_{f}=\Delta \mu_{m}=\Delta \mu ={\overset{-}{G}}_{p^{\prime }}^{\prime }-\mu .$$

For equation (21), the extra terms we need to define are $\beta_{G^{\prime }G}S$ for males and females, and $\sigma_{w\delta •G}$**:**

$$\begin{array}{c} \beta_{G_{f}^{\prime }G}S=(\sigma_{GG_{f}^{\prime }}/\sigma_{G}^{2})S\\ =(\sigma_{GG_{f}^{*}}+\sigma_{G\delta })S/\sigma_{G}^{2}\\ =\beta_{G_{f}^{*}G}S+\sigma_{G\delta }\sigma_{Gw}/\sigma_{G}^{2}\\ =\frac{1}{2}p_{1}p_{2}t(\alpha_{f}+\alpha_{m})(\alpha_{f}-\frac{1}{2}ap_{1}p_{2}t(k_{1}+k_{2})(\alpha_{f}+\alpha_{m})/\overset{-}{\varphi })/\overset{-}{\varphi }, \end{array}$$ (52)

$$\beta_{G_{m}^{\prime }G}S=\frac{1}{2}p_{1}p_{2}t(\alpha_{f}+\alpha_{m})(\alpha_{m}-\frac{1}{2}ap_{1}p_{2}t(k_{1}+k_{2})(\alpha_{f}+\alpha_{m})/\overset{-}{\varphi })/\overset{-}{\varphi }$$ (53)

and

$$\begin{array}{c} \sigma_{w\delta •G}=\sigma_{w\delta }-\sigma_{Gw}\sigma_{G\delta }/\sigma_{G}^{2}\\ =0 \end{array}$$ (54)

and as expected, the sum of equation (21) components for females and males is

$$\Delta \mu_{f}=\Delta \mu_{m}=\Delta \mu .$$

## References

[bib1] Dai F. Weeks D. E., 2006 Ordered genotypes: An extended ITO method and a general formula for genetic covariance. Am. J. Hum. Genet. 78: 1035–1045. 16685653 10.1086/504045 PMC1474083

[bib2] de Koning D.-J. Rattink A. P. Harlizius B. Groenen M. A. M. Brascamp E. W., 2001 Detection and characterization of quantitative trait loci for growth and reproduction traits in pigs. Livest. Prod. Sci. 72: 185–198. 10.2527/2001.79112812x 11768109

[bib3] de Koning D.-J. Rattink A. P. Harlizius B. van Arendonk J. A. M. Brascamp E. W., 2000 Genome-wide scan for body composition in pigs reveals important role of imprinting. Proc. Natl. Acad. Sci. USA 97: 7947–7950. 10859367 10.1073/pnas.140216397 PMC16650

[bib4] de Vries A. G. Kerr R. Tier B. Long T. Meuwissen T. H. E., 1994 Gametic imprinting effects on rate and composition of pig growth. Theor. Appl. Genet. 88: 1037–1042. 24186259 10.1007/BF00220813

[bib5] DeChiara T. M. Robertson E. J. Efstratiadis A., 1991 Parental imprinting of the mouse insulin-like growth factor-II gene. Cell 64: 849–859. 1997210 10.1016/0092-8674(91)90513-x

[bib6] Engellandt T. H. Tier B., 2002 Genetic variances due to imprinted genes in cattle. J. Anim. Breed. Genet. 119: 154–165.

[bib7] Essl A. Voith K., 2002 Genomic imprinting effects on dairy- and fitness-related traits in cattle. J. Anim. Breed. Genet. 119: 182–189.

[bib8] Ewens W. J., 1979 Mathematical Population Genetics. Springer-Verlag, Berlin.

[bib9] Falconer D. S., 1985 A note on Fisher’s “average effect” and “average excess”. Genet. Res. 46: 337–347. 4092925 10.1017/s0016672300022825

[bib10] Falconer D. S. Mackay T. F. C., 1996 Introduction to Quantitative Genetics, Ed. 4 Longman, Harlow, Essex, UK.

[bib11] Fisher R. A., 1918 The correlation between relatives on the supposition of Mendelian inheritance. Trans. R. Soc. Edinb. 52: 399–433.

[bib12] Fisher R. A., 1941 Average excess and average effect of a gene substitution. Ann. Eugen. 11: 53–63.

[bib13] Goodnight C. J., 2000 Quantitative trait loci and gene interaction: the quantitative genetics of metapopulations. Heredity 84: 587–598. 10849084 10.1046/j.1365-2540.2000.00698.x

[bib14] Hager R. Cheverud J. M. Wolf J. B., 2009 Relative contribution of additive, dominance, and imprinting effects to phenotypic variation in body size and growth between divergent selection lines of mice. Evolution 63: 1118–1128. 19187253 10.1111/j.1558-5646.2009.00638.x PMC3846273

[bib15] Harris D. L., 1964 Genotypic covariances between inbred relatives. Genetics 50: 1319–1348. 14239792 10.1093/genetics/50.6.1319 PMC1210738

[bib16] Heywood J. S., 2005 An exact form of the breeder’s equation for the evolution of a quantitative trait under natural selection. Evolution 59: 2287–2298. 16396170

[bib17] Hirooka H. de Koning D.-J. Harlizius B. van Arendonk J. A. M. Rattink A. P., 2001 A whole-genome scan for quantitative trait loci affecting teat number in pigs. J. Anim. Sci. 79: 2320–2326. 11583418 10.2527/2001.7992320x

[bib18] Kaiser, C. J., M. E. Goddard, and A. Reverter, 1998 Analysis of gametic imprinting effects for test day milk yield in Australian Holstein cattle. Proceedings of the 6th World Congress on Genetics Applied to Livestock Production, Armidale, NSW, Australia, Vol. 23, pp. 355–358.

[bib19] Knott S. A. Marklund L. Haley C. S. Andersson K. Davies W., 1998 Multiple marker mapping of quantitative trait loci in a cross between outbred wild boar and large white pigs. Genetics 149: 1069–1080. 9611214 10.1093/genetics/149.2.1069 PMC1460207

[bib20] Lee H. K. Lee S. S. Kim T. H. Jeon G. J. Jung H. W., 2003 Detection of imprinted Quantitative Trait Loci (QTL) for growth traits in pigs. Asian-Australas. J. Anim. Sci. 16: 1087–1092.

[bib21] Li C. C. Sacks L., 1954 The derivation of joint distribution and correlation between relatives by the use of stochastic matrices. Biometrics 10: 347–360.

[bib22] Luedi P. P. Dietrich F. S. Weidman J. R. Bosko J. M. Jirtle R. L., 2007 Computational and experimental identification of novel human imprinted genes. Genome Res. 17: 1723–1730. 18055845 10.1101/gr.6584707 PMC2099581

[bib23] Lynch M. Walsh B., 1998 Genetics and Analysis of Quantitative Traits. Sinauer Associates, Sunderland, MA.

[bib24] Milan D. Bidanel J.-P. Iannuccelli N. Riquet J. Amigues Y., 2002 Detection of quantitative trait loci for carcass composition traits in pigs. Genet. Sel. Evol. 34: 705–728. 12486399 10.1186/1297-9686-34-6-705 PMC2705445

[bib25] Morison I. M. Ramsay J. P. Spencer H. G., 2005 A census of mammalian imprinting. Trends Genet. 21: 457–465. 15990197 10.1016/j.tig.2005.06.008

[bib26] Nagylaki T. Lou Y., 2007 Evolution under multiallelic migration–selection models. Theor. Popul. Biol. 72: 21–40. 17470373 10.1016/j.tpb.2007.02.005

[bib27] Naumova A. K. Croteau S., 2004 Mechanisms of epigenetic variation: polymorphic imprinting. Curr. Genomics 5: 417–429.

[bib28] O’Neill M. J. Ingram R. S. Vrana P. B. Tilghman S. M., 2000 Allelic expression of IGF2 in marsupials and birds. Dev. Genes Evol. 210: 18–20. 10603082 10.1007/pl00008182

[bib29] Price G. R., 1970 Selection and Covariance. Nature 227: 520–521. 5428476 10.1038/227520a0

[bib30] Price G. R., 1972 Extension of covariance selection mathematics. Ann. Hum. Genet. 35: 485–490. 5073694 10.1111/j.1469-1809.1957.tb01874.x

[bib31] Quintanilla R. Milan D. Bidanel J.-P., 2002 A further look at quantitative trait loci affecting growth and fatness in a cross between Mieshan and Large White pig populations. Genet. Sel. Evol. 34: 193–210. 12081807 10.1186/1297-9686-34-2-193 PMC2705427

[bib32] Rand E. Cedar H., 2003 Regulation of imprinting: A multi-tiered process. J. Cell. Biochem. 88: 400–407. 12520543 10.1002/jcb.10352

[bib33] Rattink A. P. de Koning D. J. Faivre M. Harlizius B. van Arendonk J. A. M., 2000 Fine mapping and imprinting analysis for fatness trait QTLs in pigs. Mamm. Genome 11: 656–661. 10920235 10.1007/s003350010117

[bib34] Sandovici I. Kassovska-Bratinova S. Loredo-Osti J. C. Leppert M. Suarez A., 2005 Interindividual variability and parent of origin DNA methylation differences at specific human Alu elements. Hum. Mol. Genet. 14: 2135–2143. 15972727 10.1093/hmg/ddi218

[bib35] Sandovici I. Leppert M. Hawk P. R. Suarez A. Linares Y., 2003 Familial aggregation of abnormal methylation of parental alleles at the IGF2/H19 and IGF2R differentially methylated regions. Hum. Mol. Genet. 12: 1569–1578. 12812984 10.1093/hmg/ddg167

[bib36] Santure A. W. Spencer H. G., 2006 Influence of mom and dad: Quantitative genetic models for maternal effects and genomic imprinting. Genetics 173: 2297–2316. 16751674 10.1534/genetics.105.049494 PMC1569723

[bib37] Schaeffer L. R. Kennedy B. W. Gibson J. P., 1989 The inverse of the gametic relationship matrix. J. Dairy Sci. 72: 1266–1272.

[bib38] Spencer H. G., 2002 The correlation between relatives on the supposition of genomic imprinting. Genetics 161: 411–417. 12019254 10.1093/genetics/161.1.411 PMC1462108

[bib39] Stella A. Stalder K. J. Saxton A. M. Boettcher P. J., 2003 Estimation of variances for gametic effects on litter size in Yorkshire and Landrace swine. J. Anim. Sci. 81: 2171–2178. 12968691 10.2527/2003.8192171x

[bib40] Templeton A. R., 1987 The general relationship between average effect and average excess. Genet. Res. 49: 69–70. 3569910 10.1017/s0016672300026756

[bib41] Tier B. Solkner J., 1993 Analysing gametic variation with an animal model. Theor. Appl. Genet. 85: 868–872. 24196062 10.1007/BF00225031

